# Comparative Efficacy of Combined Radiotherapy, Systemic Therapy, and Androgen Deprivation Therapy for Metastatic Hormone-Sensitive Prostate Cancer: A Network Meta-Analysis and Systematic Review

**DOI:** 10.3389/fonc.2020.567616

**Published:** 2020-10-20

**Authors:** Yuhan Wang, Huiming Gui, Juan Wang, Junqiang Tian, Hanzhang Wang, Chaozhao Liang, Zongyao Hao, Ronald Rodriguez, Zhiping Wang

**Affiliations:** ^1^Key Laboratory of Urological Diseases in Gansu Province, Department of Urology, Gansu Nephro-Urological Clinical Center, Lanzhou University Second Hospital, Lanzhou, China; ^2^Department of Urology, The University of Texas Health Science Center at San Antonio, San Antonio, TX, United States; ^3^Department of Urology, The First Affiliated Hospital of Anhui Medical University, Hefei, China

**Keywords:** mHSPC, radiotherapy, chemotherapy, local therapy, indirect comparisons

## Abstract

**Background:** Recent randomized clinical trials have examined the efficacy of different combinations of systemic and local treatment approaches for metastatic hormone-sensitive prostate cancer (mHSPC). We compared the efficacy of these combined regimens in order to identify the optimal therapy for specific patient subgroups.

**Methods:** The treatments were abiraterone (ABI), apalutamide (APA), docetaxel (DOC), enzalutamide (ENZ), and radiotherapy (RT) combined with androgen-deprivation therapy (ADT). Five electronic databases were searched up to May 7, 2020 for relevant trials. The risk of bias in the included trials was evaluated with the Cochrane tool. The hazard ratio (HR) with 95% confidence interval (CI) was determined for the included trials and indirect comparisons were performed using the R software.

**Results:** In total, 10 randomized, controlled trials with 11,194 patients were included in the meta-analysis. ADT + RT was superior to ADT monotherapy in terms of overall survival (HR = 0.96, 95% CI: 0.85–1.1) and conferred a survival benefit in a subgroup of low-volume patients (HR = 0.68, 95% CI: 0.54–0.87). Combined systemic treatments were significantly superior to ADT monotherapy in comparisons of survival and prostate-specific antigen response, including in the high-volume subgroup; meanwhile, in the low-volume subgroup only ADT + ENZ (HR = 0.38, 95% CI 0.21–0.69) showed a significant clinical benefit. In the Gleason score <8 subgroup, all combined systemic treatments were superior to ADT monotherapy, but the results were only significant for ADT + APA (HR = 0.56, 95% CI: 0.33–0.95) and ADT + DOC (HR = 0.71, 95% CI: 0.54–0.92). In the Gleason score ≥8 subgroup, ADT monotherapy was inferior (albeit not significantly) to combined treatments. In a ranking of performed comparisons, ADT + ENZ was the optimal regimen, although this was non-significant. Combined therapies also demonstrated superiority in quality-of-life indicators such as time to skeletal events and pain progression.

**Conclusion:** ADT + radiotherapy led to superior outcomes in mHSPC patients with low-volume disease. While all combined systemic regimens confer a survival advantage over ADT monotherapy, the optimal treatment approach for certain mHSPC patient subgroups remains to be determined.

## Introduction

Prostate cancer (PC) is the most common cancer type in senior males worldwide (1). In the last few decades, androgen deprivation therapy (ADT) has been the standard of care for metastatic PC. ADT blocks tumor growth induced by androgen, thereby improving the prognosis of patients. However, the rapid progression to castration resistance in patients treated with ADT necessitates more effective treatment options.

The therapeutic armamentarium for metastatic hormone-sensitive (mHSPC) patients has rapidly expanded with the development of novel treatments such as an androgen synthesis inhibitor, chemotherapy, and next-generation antiandrogen drugs, which have greatly improved the efficacy of ADT. The STAMPEDE trial demonstrated that combining docetaxel (DOC) or abiraterone (ABI) with ADT conferred a clinically meaningful survival advantage in patients with mHSPC (2, 3). Meanwhile, results from the TITAN, ENZAMET, and ARCHES trials revealed a survival advantage for mHSPC patients when either enzalutamide (ENZ) or apalutamide (APA) was combined with ADT (4–6). Based on the encouraging results from these trials, the National Comprehensive Cancer Network guidelines has included combined treatments as first-line therapy for mHSPC (7). In addition to systemic therapies, local treatment of the prostate combined with ADT was also shown to prolong survival in mHSPC patients. Results from two randomized clinical trials revealed a potential survival benefit with local radiotherapy (RT) complementary to ADT in certain subgroups of mHSPC patients (8, 9). At the 2019 Advanced PC Consensus Conference, 98% of panelists believed that local treatment can enhance survival in patients with low-volume metastatic PC (10). Although many combined treatment approaches have been shown to be effective, there are no data on the comparative efficacy of systemic and local therapies combined with ADT.

To address this issue, in this study we indirectly compared different combined treatment approaches (ADT + ABI + prednisolone [AAP], ADT + APA, ADT + docetaxel (DOC), ADT + ENZ, ADT + RT, and ADT monotherapy) in order to identify an optimal treatment strategy for prolonging survival and improving quality of life (QoL) in mHSPC patients. We also performed subgroup analyses of patients with different disease volumes and Gleason scores.

## Materials and Methods

### Literature Search Strategy

The systematic review was performed according to the Preferred Reporting Items for Systematic Reviews and Meta-Analysis guidelines, and the protocol was registered a priori in the PROSPERO database. Computerized literature databases (PubMed/MEDLINE, EMBASE, Cochrane library, and clinicaltrials.gov) and gray literature (American Society of Clinical Oncology) were searched to identify all relevant publications up to May 7, 2020. The complete search strategy is described in Supplementary Material 1. References cited in selected articles as well as relevant reviews were manually searched to ensure the completeness of our analysis.

### Inclusion Criteria

#### Participants

We included studies that enrolled patients with mHSPC aged ≥18 years. Studies of patients with localized or castration-resistant PC were excluded.

#### Study Design

Only phase 3 randomized controlled trials (RCTs) were retained; retrospective, prospective, and non-randomized studies were excluded.

#### Types of Intervention

We focused on the following eight interventions: ADT monotherapy, ADT + APA, ADT + AAP, ADT + DOC, ADT + ENZ, and ADT + RT.

#### Outcomes

Outcomes of interest were overall survival (OS), prostate-specific antigen progression-free survival (PSA-PFS), time to symptomatic skeletal events (SSE), time to pain progression, and time to chemotherapy. OS was defined as the time from randomization to death for any reason. PSA-PFS was measured as the interval from randomization to the earliest event of PSA progression according to the PC Working Group 2 criteria (11). Time to SSE was defined as the time from randomization to any of the following skeletal-related events: bone radiation treatment or surgery, clinically apparent pathologic bone fracture, or spinal cord compression. Time to pain progression was defined as the time from randomization to the first increase of ≥30% from the baseline in the Brief Pain Inventory–Short Form score. Time to chemotherapy was defined as time from randomization to initiation of chemotherapy for PC.

Only articles published in English were included. Titles and abstracts were screened for initial study inclusion. The full texts of potential articles were thoroughly examined when the title and abstract were insufficient to determine whether the study met the inclusion criteria. In instances where there was more than one publication resulting from the same clinical trial, we selected the most complete publication for analysis. The detailed PRISMA flow graph of the inclusion process is presented in Supplementary Material 2.

### Data Extraction and Quality Assessment

Two investigators independently extracted the data from the included studies. The following information was obtained from the publications: author, year of publication, sample size, interventions, and main outcome measures defined in our inclusion criteria. We also extracted subgroup outcome data from each study (e.g., disease volume and Gleason score). As in the CHAARTED study, high-volume disease was defined as the presence of visceral metastases or ≥4 bone lesions with ≥1 lesion beyond the vertebral bodies and pelvis (12).

The methodologic quality of each study was assessed with the Cochrane Collaboration tool by two independent researchers. Aspects such as randomization, allocation, and blinding processes, outcome reporting, and other types of bias were assessed, with each one assigned a grade of low, high, or uncertain risk based on the result of the quality evaluation. Any disagreement was resolved by discussion with a third reviewer until a consensus was reached.

### Data Synthesis and Analysis

Indirect comparisons were performed based on the Bayesian framework model using the “gemtc” package of the R 3.5.3 software (13, 14). OS was estimated using the hazard ratio (HR) with 95% confidence interval (CI). A 2-tailed *p* < 0.05 was considered statistically significant. Based on the deviance information criterion (DIC) value, a fixed-, or random-effects model was applied to the Bayesian framework model for data analysis. The convergence of the models was assessed with Brooks-Gelman-Rubin, trace, and density plots. A funnel plot was used to assess publication bias. Rank probabilities were calculated to determine the hierarchy of treatments. The “mtc.anohe” command in the “gemtc” package was used to evaluate global heterogeneity, which was documented with the variance parameter *I*^2^; a value >50% was considered indicative of significant heterogeneity. The geometry of the treatment network was established using the R software. Inconsistency was assessed if any closed loop existed in the network. The following comparisons of treatments were performed: ADT monotherapy, ADT + APA, ADT + AAP, ADT + DOC, ADT + ENZ, and ADT + RT for OS and PSA-PFS; ADT + APA, ADT + AAP, ADT + ENZ, and ADT monotherapy for time to SSE and time to pain progression; and ADT + APA, ADT + AAP, and ADT monotherapy for time to chemotherapy.

## Results

### Baseline Characteristics of Included Studies

**Figure 1:**
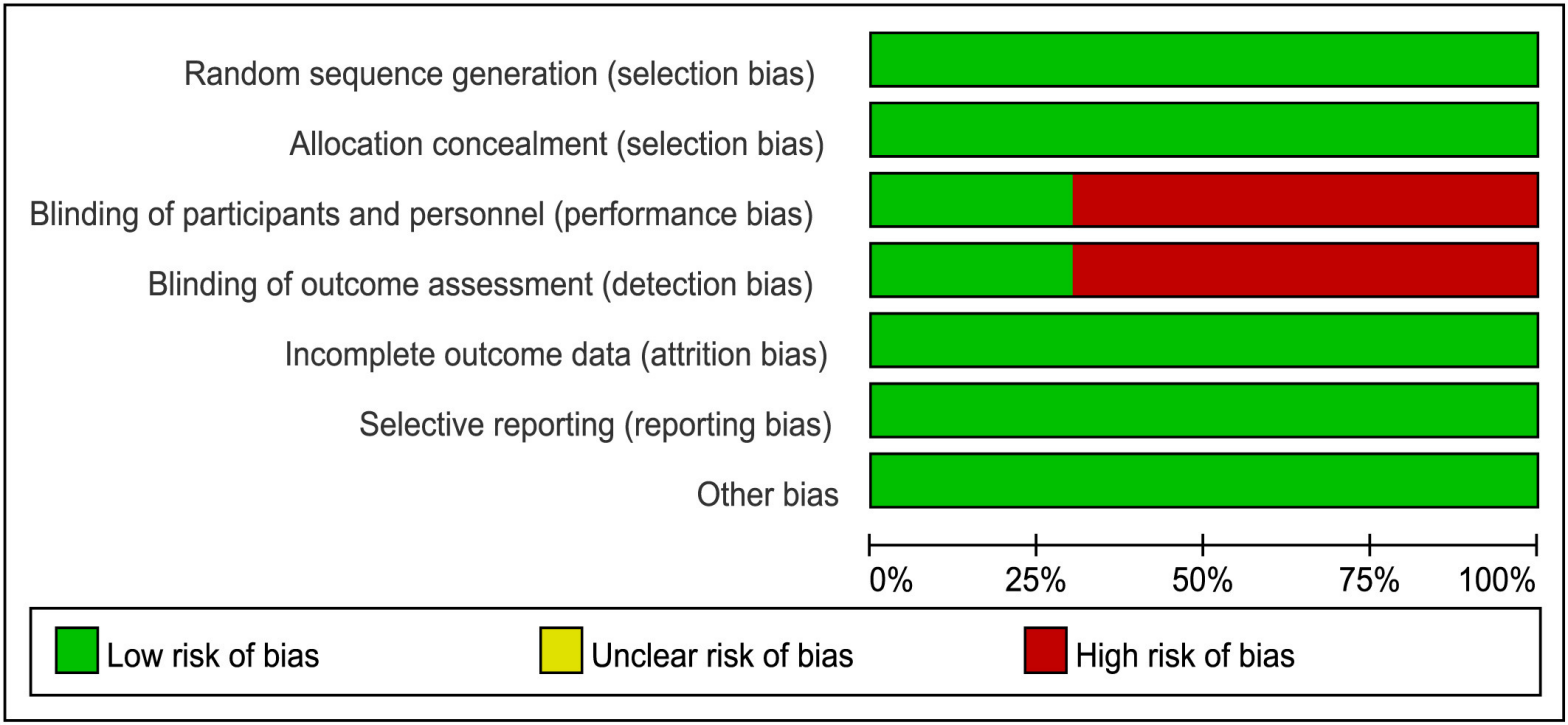
Judgment of risk of bias for each item presented as percentages across all eligible studies.

**Figure 2:**
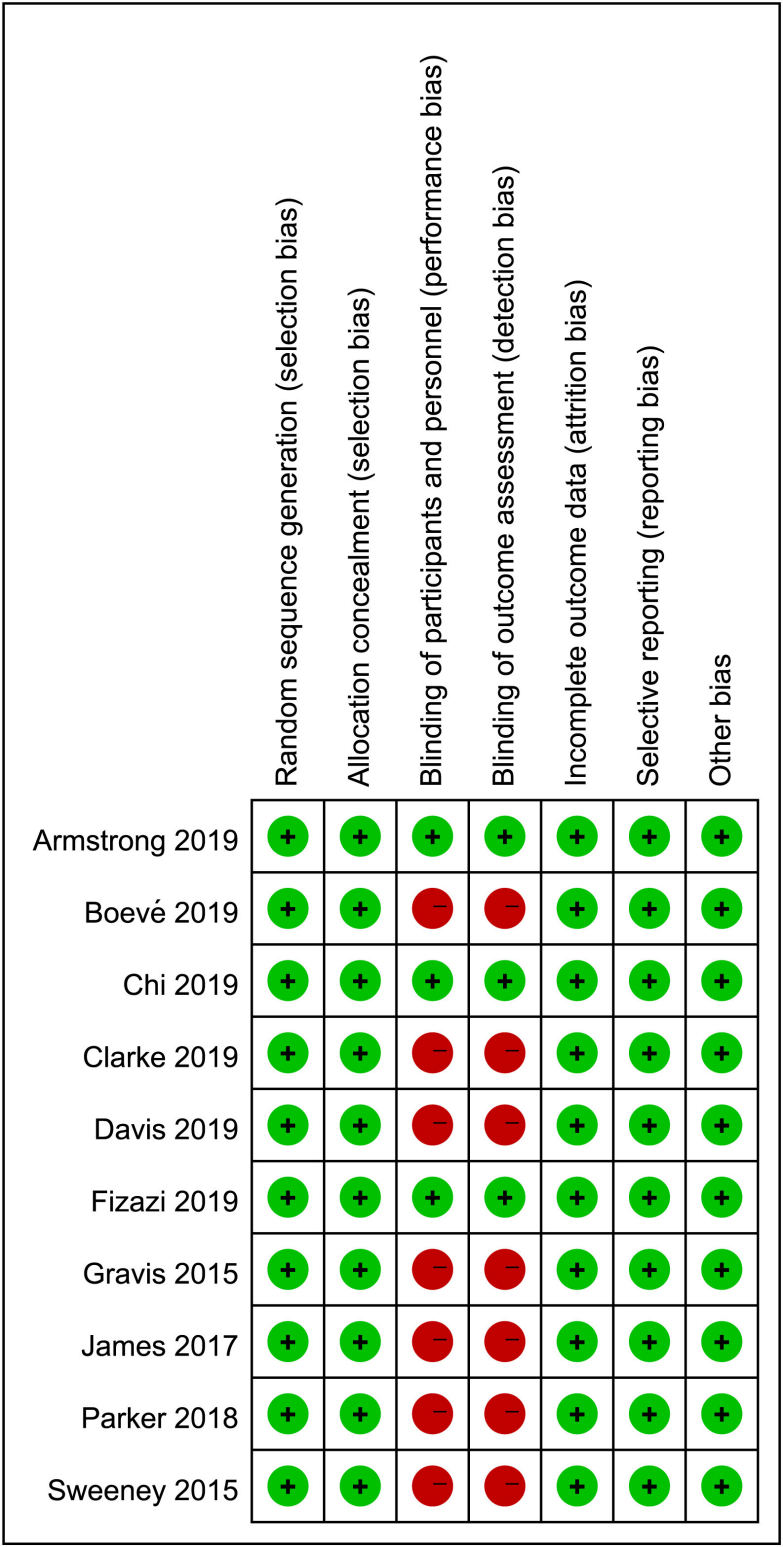
Reviewers' judgments of each risk of bias item for eligible studies.

The literature search returned of 639 records. After removing duplicates, 516 publications were retained for title and abstract screening, and 15 were selected for full-text assessment. Ultimately, 10 RCTs with 11,194 participants were included in our analysis. The baseline characteristics of the included studies are listed in Table 1. Although we assumed that the transitivity assumption was satisfied in our study, this was difficult to validate owing to the paucity of the baseline data in several of the included trials. The quality of each included article was evaluated using the Cochrane Collaboration tool; the results of quality assessment are summarized in Figures 1, 2. Seven out of 10 studies were open-label trials and lacked blinding in the study design. All other aspects of the selected articles were determined to be of high quality according to our assessment.

**Table 1:** Characteristics of included randomized clinical trials in this analysis.

**References**	**Study name**	**Samples (experiment/control)**	**Intervention (experiment group)**	**Intervention (control group)**	**Primary outcome**
Chi et al. (5)	TITAN	525/527	ADT + Apalutamide (240 mg/d)	ADT	OS, rPFS
James et al. (2)	STAMPEDE-G arm	960/957	ADT + Abiraterone (1,000 mg/d) + Prednisolone (5 mg/d)	ADT	OS, FFS
Fizazi et al. (15)	LATITUDE	597/602	ADT + Abiraterone (1,000 mg/d) + Prednisolone (5 mg/d)	ADT	OS, rPFS
Gravis et al. (16)	GETUG-AFU-15	192/193	ADT + Docetaxel (75 mg/m^2^ for 21 d, up to 9 cycles)	ADT	OS
Sweeney et al. (12)	CHAARTED	397/393	ADT + Docetaxel (75 mg/m^2^ for 21 d, up to 9 cycles)	ADT	OS
Clarke et al. (17)	STAMPEDE-C arm	592/1,184	ADT + Docetaxel (75 mg/m^2^ for 21 d, up to 6-cycle)	ADT	OS
Davis et al. (4)	ENZAMET	563/562	ADT + Enzalutamide (160 mg/d)	ADT	OS, PFS
Armstrong et al. (6)	ARCHES	574/576	ADT + Enzalutamide (160 mg/d)	ADT	rPFS
Boevé et al. (8)	HORRAD	216/216	ADT + external beam radiation therapy	ADT	OS
Parker et al. (9)	STAMPEDE-H arm	1,032/1,029	ADT + external beam radiation therapy	ADT	OS, FFS

*OS, Overall survival; rPFS, radiographic progression-free survival; PFS, progression-free survival; FFS, failure-free survival; bPFS, biochemical progression-free survival; ADT, androgen deprivation therapy*.

**Figure 3:**
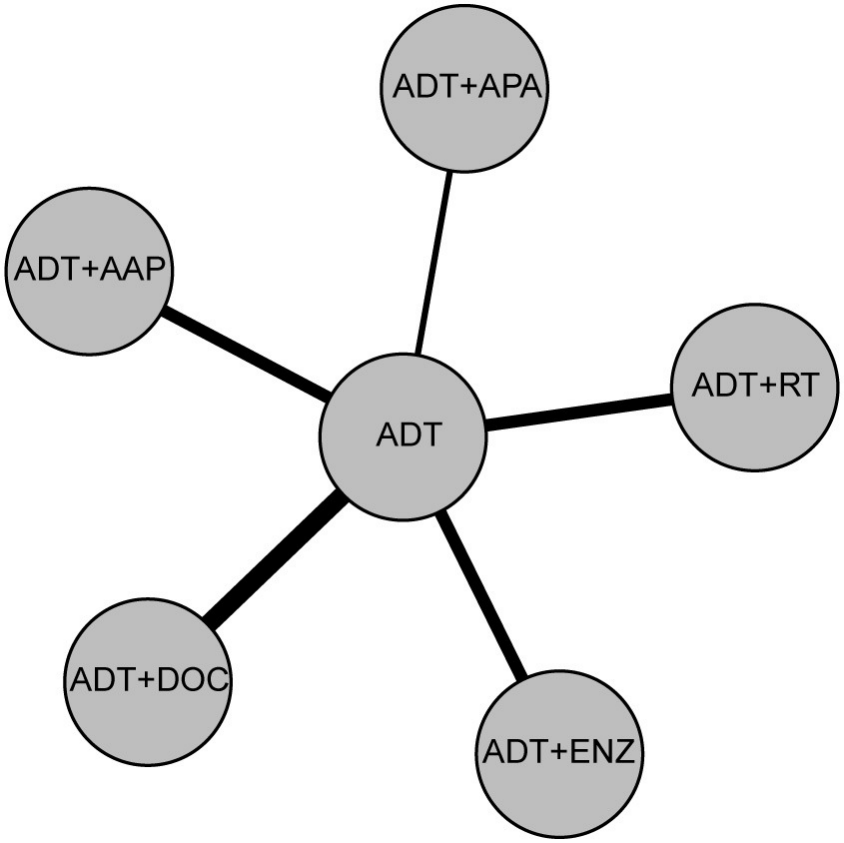
Network plot of six treatment modalities. For OS, PSA-PFS, and Gleason score <8/≥8, low/high volume subgroups, ADT monotherapy, ADT + APA, ADT + AAP, ADT + DOC, ADT + ENZ, and ADT+RT were compared. For time to SSE and time to pain progression, ADT + APA, ADT + AAP, ADT + ENZ, and ADT monotherapy were compared. For time to chemotherapy, ADT + APA, ADT + AAP, and ADT monotherapy were compared.

ADT monotherapy, ADT + APA, ADT + AAP, ADT + DOC, ADT + ENZ, and ADT + RT were compared in our study. The treatment network is shown in Figure 3, with the thickness of each line in the network plot proportional to the number of comparisons. Based on the DIC value, the random-effects model was applied to the analysis of PSA-PFS, time to SSE, and OS in the Gleason score ≥8 subgroup; the fixed-effects model was applied to the other comparisons.

### Indirect Comparisons of OS

**Figure 4:**
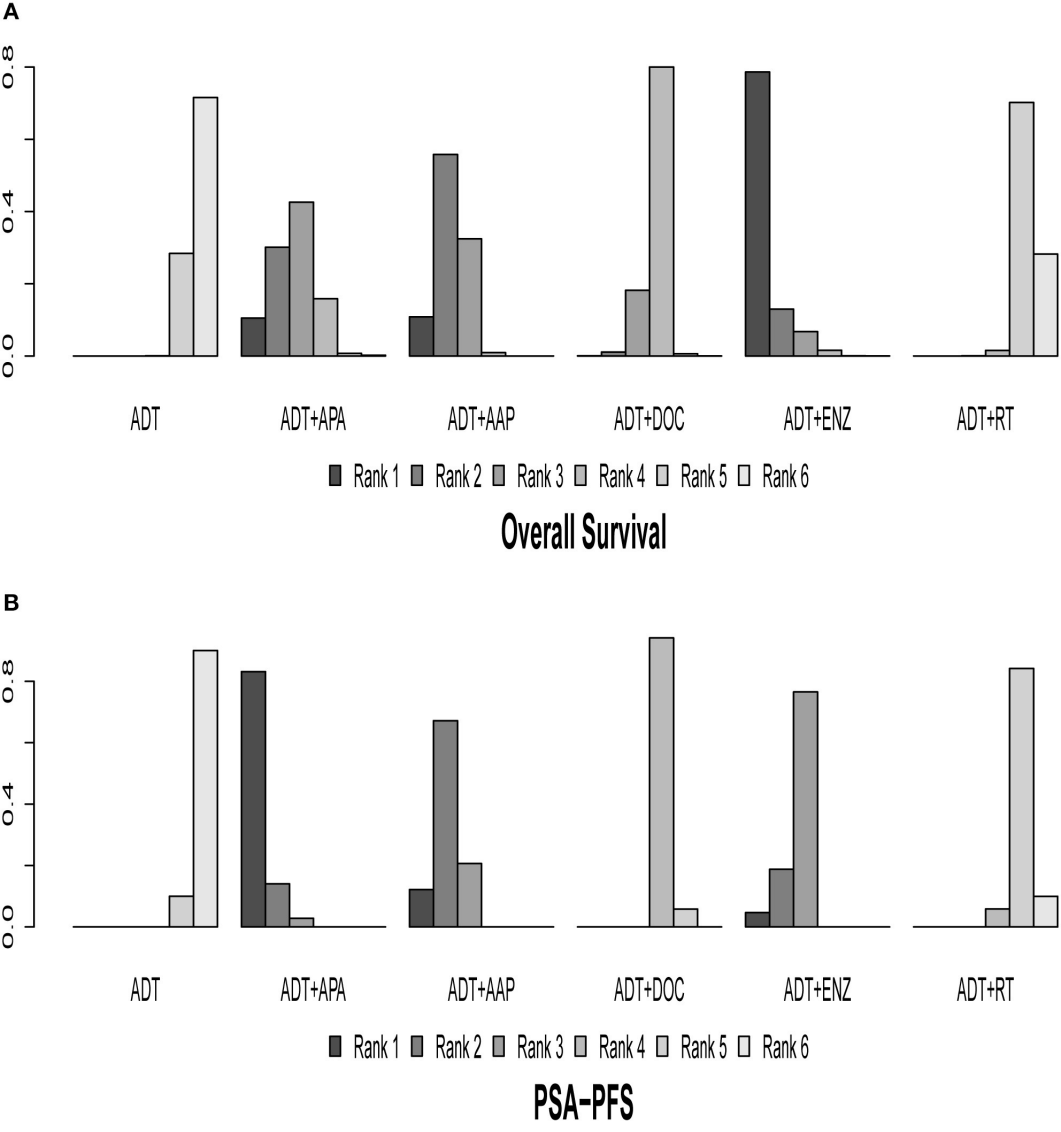
Ranking plot based on the probabilities of interventions in the overall population. **(A)** OS. **(B)** PSA-PFS. The height of each column reflects the probability of the rank.

The results of indirect comparisons are shown in Table 2. Compared to ADT monotherapy, ADT + AAP (HR = 0.64, 95% CI 0.56–0.73), ADT + APA (HR = 0.67, 95% CI 0.51–0.89), ADT + DOC (HR = 0.78, 95% CI 0.69–0.88), and ADT + ENZ (HR = 0.53, 95% CI 0.37–0.75) all showed statistically significant survival benefits, while no advantages were observed in comparisons between the four combined treatment regimens. ADT + RT had the highest HR compared to ADT monotherapy (HR = 0.96, 95% CI 0.85–1.1) and was inferior to every combined systemic therapy. Ranking results indicated a high likelihood that ADT + ENZ was superior (78.58%) to the other regimens in prolonging OS (Figure 4, Table 3).

**Table 2:** The meta-analysis results of all comparisons.

	**ADT + APA** **vs.** **ADT**	**ADT + AAP** **vs.** **ADT**	**ADT + DOC** **vs.** **ADT**	**ADT + ENZ** **vs.** **ADT**	**ADT + RT** **vs.** **ADT**	**ADT + AAP** **vs.** **ADT + APA**	**ADT + DOC** **vs.** **ADT + APA**	**ADT + ENZ** **vs.** **ADT + APA**	**ADT + RT** **vs.** **ADT + APA**	**ADT + DOC** **vs.** **ADT + AAP**	**ADT + ENZ** **vs.** **ADT + AAP**	**ADT + RT** **vs.** **ADT + AAP**	**ADT + ENZ** **vs.** **ADT + DOC**	**ADT + RT** **vs.** **ADT + DOC**	**ADT + RT** **vs.** **ADT + ENZ**
OS [HR (95%CI)]	**0.67** ** (0.51, 0.89)**	**0.64** ** (0.56, 0.73)**	**0.78** ** (0.69, 0.88)**	**0.53** ** (0.37, 0.75)**	0.96 (0.85, 1.1)	0.95 (0.70, 1.30)	1.20 (0.85, 1.60)	0.78 (0.50, 1.2)	**1.4** ** (1.1, 1.9)**	1.20 (1.0, 1.50)	0.82 (0.57, 1.2)	**1.5** ** (1.3, 1.8)**	**0.68** ** (0.47, 0.99)**	1.2 (1.0, 1.5)	**1.8** ** (1.3, 2.7)**
PSA-PFS	**0.26** ** (0.21, 0.32)**	**0.30** ** (0.26, 0.35)**	**0.67** ** (0.54, 0.84)**	**0.34** ** (0.26, 0.44)**	0.86 (0.69, 1.1)	1.2 (0.9, 1.5)	**2.6** ** (1.9, 3.5)**	1.3 (0.94, 1.8)	**3.3** ** (2.4, 4.5)**	**2.2** ** (1.7, 2.9)**	1.1 (0.84, 1.5)	**2.9** ** (2.2, 3.7)**	**0.50** ** (0.36, 0.71)**	1.3 (0.94, 1.8)	**2.6** ** (1.8, 3.6)**
Time to SSE	0.80 (0.33, 2)	0.76 (0.32, 1.8)	NA	0.51 (0.20, 1.3)	NA	0.94 (0.27, 3.3)	NA	0.64 (0.17, 2.3)	NA	NA	0.68 (0.19, 2.4)	NA	NA	NA	NA
Time to pain progression	0.83 (0.65, 1.1)	**0.72** ** (0.61, 0.86)**	NA	0.91 (0.78, 1.1)	NA	0.88 (0.65, 1,2)	NA	1.1 (0.83, 1.5)	NA	NA	1.3 (1.0,1.6)	NA	NA	NA	NA
Time to chemotherapy	**0.39** ** (0.27, 0.56)**	**0.51** ** (0.41, 0.63)**	NA	NA	NA	1.3 (0.85, 2)	NA	NA	NA	NA	NA	NA	NA	NA	NA
OS for high-volume subgroup	**0.68** ** (0.50, 0.92)**	**0.62** ** (0.50, 0.74)**	**0.73** ** (0.62, 0.86)**	**0.64** ** (0.42, 0.99)**	1.1 (0.92, 1.2)	0.91 (0.64, 1.3)	1.1 (0.76, 1.5)	0.95 (0.56, 1.6)	**1.6** ** (1.1, 2.2)**	1.2 (0.93, 1.5)	1.0 (0.65, 1.7)	**1.7** ** (1.4, 2.2)**	0.88 (0.56, 1.4)	**1.5** ** (1.2, 1.8)**	**1.7** ** (1.1, 2.6)**
OS for low-volume subgroup	0.67 (0.34, 1.30)	0.72 (0.47, 1.1)	0.81 (0.64, 1.0)	**0.38** ** (0.21, 0.69)**	**0.68** ** (0.54, 0.87)**	1.1 (0.49, 2.4)	1.2 (0.59, 2.5)	0.57 (0.23, 1.4)	1.0 (0.50, 2.1)	1.1 (0.69, 1.8)	0.53 (0.25, 1.1)	0.95 (0.58, 1.5)	**0.47** ** (0.25, 0.89)**	0.84 (0.60, 1.2)	1.8 (0.95, 3.4)
OS for GS<8 subgroup	**0.56** ** (0.33, 0.95)**	0.44 (0.15, 1.3)	**0.71** ** (0.54, 0.92)**	0.59 (0.30, 1.2)	1.1 (0.84, 1.5)	0.78 (0.23, 2.6)	1.3 (0.70, 2.3)	1.1 (0.45, 2.5)	**2.0** ** (1.1, 3.7)**	1.6 (0.54, 4.8)	1.4 (0.38, 4.8)	2.6 (0.85, 7.7)	0.83 (0.4, 1.7)	**1.6** ** (1.1, 2.4)**	1.9 (0.92, 4.0)
OS for GS ≥8 subgroup	0.73 (0.36, 1.5)	0.67 (0.35, 1.3)	0.78 (0.53, 1.2)	0.70 (0.35, 1.4)	0.90 (0.48, 1.7)	0.92 (0.35, 2.4)	1.1 (0.48, 2.4)	0.95 (0.35, 2.5)	1.2 (0.48, 3.2)	1.2 (0.54, 2.5)	1.0 (0.4, 2.6)	1.4 (0.55, 3.4)	0.89 (0.40, 2)	1.2 (0.55, 2.5)	1.3 (0.5, 3.4)

*OS, Overall survival; HR, hazard ratio; 95%CI, 95% confidence intervals; PSA-PFS, prostate specific antigen progression-free survival; SSE, symptomatic skeletal events; GS, Gleason score; ADT, androgen deprivation therapy; APA, apalutamide; AAP, abiraterone and prednisolone; DOC, docetaxel; ENZ, enzalutamide; RT, radiotherapy; NA, not available. Comparison in bold refers to the statistically significant comparison*.

**Table 3:** Ranking results of all comparisons.

	**Ranks**	**ADT**	**ADT + APA**	**ADT + AAP**	**ADT + DOC**	**ADT + ENZ**	**ADT + RT**
OS [HR (95%CI)]	Rank 1	0	10.36	11.00	0.06	78.58	0
	Rank 2	0	30.35	55.74	1.08	12.83	0
	Rank 3	0	42.25	32.25	18.59	6.84	0.07
	Rank 4	0.09	30.94	1.01	79.52	1.66	1.78
	Rank 5	27.79	0.09	0	0.75	0.07	70.50
	Rank 6	72.12	0.02	0	0	0.02	27.65
PSA-PFS	Rank 1	0	83.17	12.17	0	4.66	0
	Rank 2	0	14.05	67.18	0	18.77	0
	Rank 3	0	2.79	20.65	0.01	76.55	0
	Rank 4	0	0	0	94.2	0.01	5.84
	Rank 5	9.97	0	0	5.83	0	84.20
	Rank 6	90.03	0	0	0.01	0	9.96
Time to SSE	Rank 1	0.62	14.56	16.54	NA	68.28	NA
	Rank 2	8.06	31.58	40.50	NA	19.86	NA
	Rank 3	34.18	30.55	27.72	NA	7.55	NA
	Rank 4	57.14	23.30	15.24	NA	4.31	NA
Time to pain progression	Rank 1	0	18.74	79.90	NA	0.136	NA
	Rank 2	0.93	56.61	18.60	NA	23.86	NA
	Rank 3	17.25	18.96	1.48	NA	62.31	NA
	Rank 4	81.82	5.69	0.02	NA	12.47	NA
Time to chemotherapy	Rank 1	0	89.31	10.69	NA	NA	NA
	Rank 2	0	10.69	89.31	NA	NA	NA
	Rank 3	1	0	0	NA	NA	NA
Low-volume	Rank 1	0	14.25	4.23	0.23	78.90	2.38
	Rank 2	0	30	22.1	4.27	15.25	28.37
	Rank 3	0.04	16.03	26.08	15.18	3.84	38.83
	Rank 4	1.42	14.74	24.04	34.76	1.39	23.65
	Rank 5	20.20	13.27	17.62	41.64	0.55	6.72
	Rank 6	78.34	11.71	5.92	3.91	0.07	0.05
High-volume	Rank 1	0	28.25	62.28	4.26	5.21	0
	Rank 2	0	32.63	29.84	25.32	12.21	0
	Rank 3	0.04	23.41	6.71	47.88	21.90	0.06
	Rank 4	6.60	14.81	1.16	22.52	52.12	2.78
	Rank 5	75.63	0.61	0	0.01	4.72	19.03
	Rank 6	17.73	0.29	0	0	3.84	78.13
GS<8	Rank 1	0	21.64	56.04	2.32	20	0
	Rank 2	0.01	37.65	17.40	14.07	30.80	0.07
	Rank 3	0.51	26.32	10.88	37.46	24.22	0.61
	Rank 4	10.67	12.52	8.95	44.90	18.08	4.88
	Rank 5	69.81	1.22	2.58	1.10	3.58	21.71
	Rank 6	19	0.65	4.15	0.14	3.32	72.73
GS≥8	Rank 1	0.03	23.79	32.98	7.79	29.79	5.63
	Rank 2	0.47	20.90	27.06	19.65	22.89	9.02
	Rank 3	3.45	18.27	18.03	29.14	17.72	13.39
	Rank 4	14.80	15.73	10.73	26.95	13.14	18.66
	Rank 5	37.26	10.32	6.02	12.07	8.39	25.95
	Rank 6	43.99	11.00	5.18	4.41	8.07	27.36

*OS, Overall survival; HR, hazard ratio; 95%CI, 95% confidence intervals; PSA-PFS, prostate specific antigen progression-free survival; GS, Gleason score; ADT, androgen deprivation therapy; APA, apalutamide; AAP, abiraterone and prednisolone; DOC, docetaxel; ENZ, enzalutamide; RT, radiotherapy; NA, not available*.

### Indirect Comparisons of PSA-PFS

Nine of the 10 trials were included in the analysis of PSA-PFS. ADT + AAP (HR = 0.30, 95% CI: 0.26–0.35), ADT + APA (HR = 0.26, 95% CI: 0.21–0.32), ADT + DOC (HR = 0.67, 95% CI: 0.54–0.84), ADT + ENZ (HR = 0.34, 95% CI: 0.26–0.44), and ADT + RT (HR = 0.86, 95% CI: 0.69–1.1) conferred a survival benefit over ADT monotherapy. ADT + APA was the most effective combined treatment regimen (83.17%) whereas ADT + RT (84.20%) ranked last. Results of indirect comparisons are shown in Table 2 and detailed ranking results are shown in Figure 4, Table 3.

### Indirect Comparisons of Health-Related QoL Outcomes

**Figure 5:**
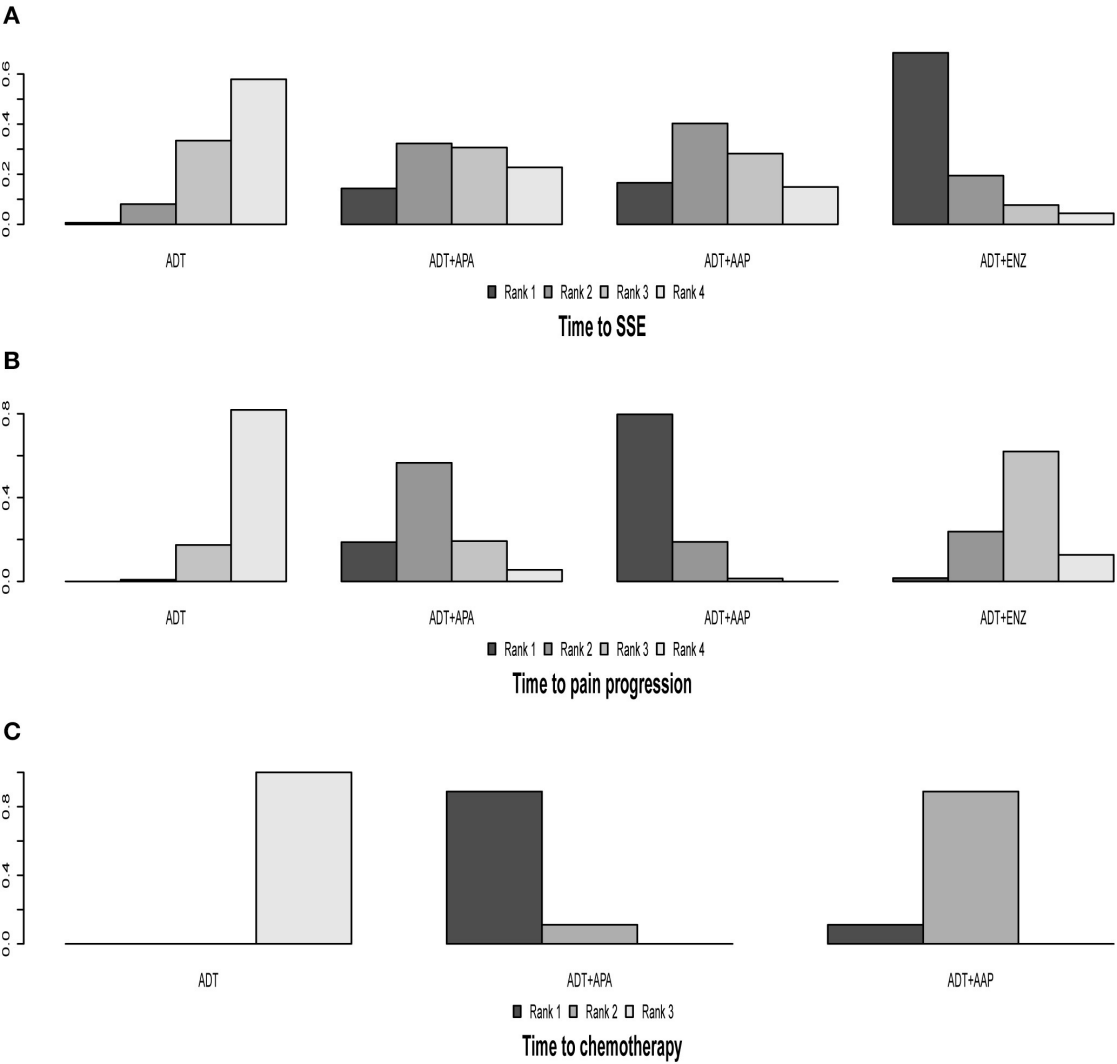
Ranking plot based on the probabilities of interventions in the analysis of secondary outcomes. **(A)** Time to SSE. **(B)** Time to pain progression. **(C)** Time to chemotherapy. The height of each column reflects the probability of the rank.

We compared two health-related QoL outcomes; the results of indirect comparisons are shown in Table 2 and detailed ranking results are shown in Figure 5, Table 3. Four regimens (ADT + APA, ADT + AAP, ADT + ENZ, and ADT monotherapy) were compared in terms of time to SSE. The combined treatments had longer times to SSE than ADT monotherapy, although the differences were not statistically significant. ADT + ENZ (HR = 0.51, 95% CI: 0.20–1.3) was the most effective regimen compared to ADT monotherapy according to rank, whereas ADT + APA had the highest HR (0.80, 95% CI: 0.33–2.0). However, these findings were not statistically significant.

The four regimens (ADT + APA, ADT + AAP, ADT + ENZ, and ADT monotherapy) were compared in terms of time to pain progression; all three combined therapies were found to be superior to ADT monotherapy but only ADT + AAP showed a statistically significant difference (HR = 0.72, 95% CI: 0.61–0.86). ADT + AAP was the highest-ranking regimen (79.90%), although all indirect comparisons yielded non-significant results.

### Indirect Comparisons of Time to Chemotherapy

Two trials (TITAN and LATITUDE) were included in the analysis. Indirect comparisons revealed that both ADT + AAP (HR = 0.51, 95% CI: 0.41–0.63) and ADT + APA (HR = 0.39, 95% CI: 0.27–0.56) prolonged the time to chemotherapy compared to ADT monotherapy (Table 2). ADT + APA ranked highest (89.31%) among the three treatment regimens in time to chemotherapy, but this result was non-significant (Figure 5, Table 3).

### Subgroup Analysis of Disease Volume

**Figure 6:**
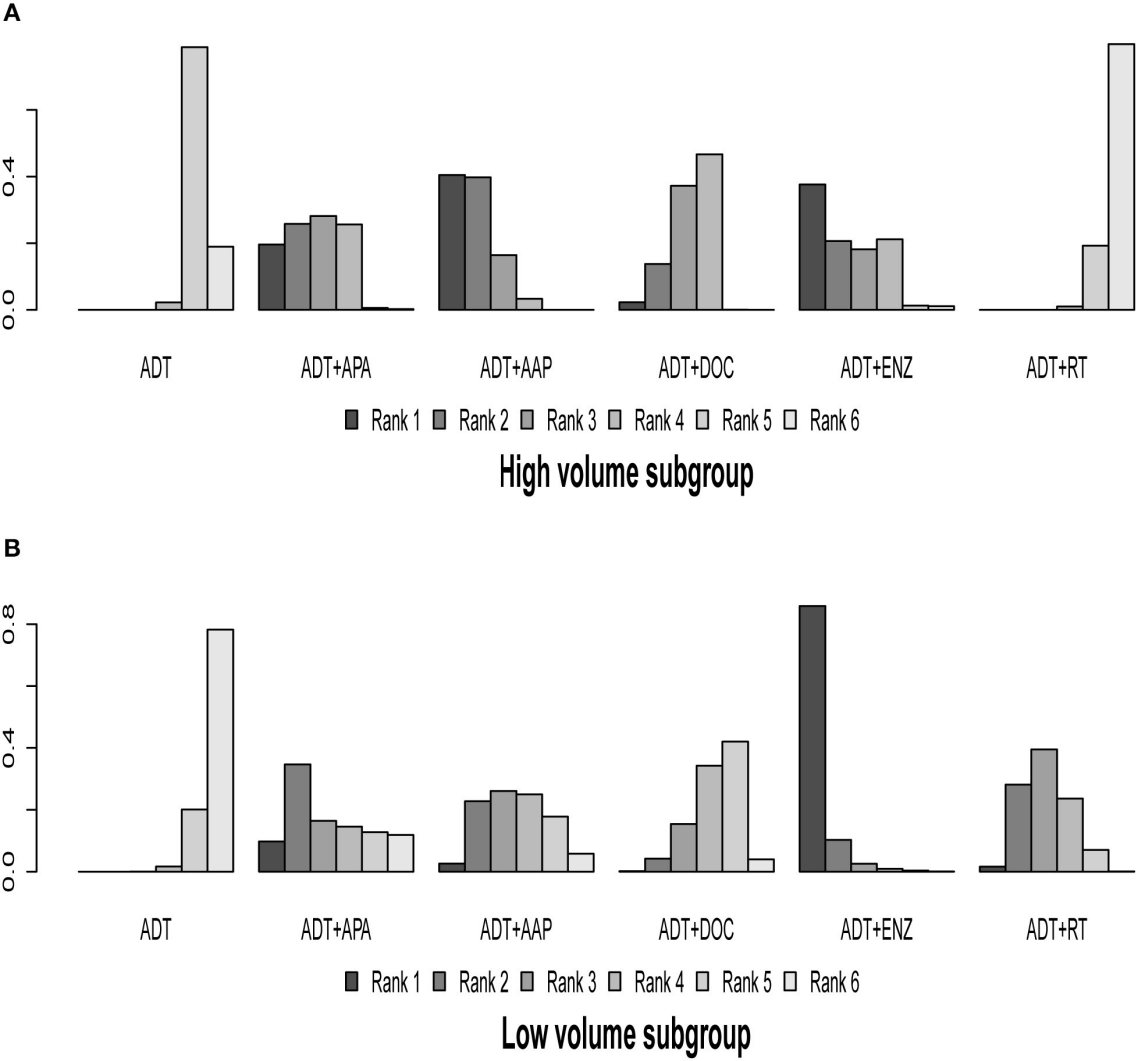
Ranking plot based on the probabilities of interventions in patients with different disease volume: **(A)** High-volume subgroup; **(B)** Low-volume subgroup. The height of every column refers to the probabilities of the rank. The actual outcome being measured in this figure was overall survival.

Eight of the 10 trials were included in this subgroup analysis; the ARCHES and STAMPEDE-ABI arm were excluded because eligible outcome data were lacking. The results of indirect comparisons are shown in Table 2 and the detailed ranking results are shown in Figure 6, Table 3.

In the high-volume disease subgroup, ADT + AAP (HR = 0.62, 95% CI: 0.50–0.74), ADT + APA (HR = 0.68, 95% CI: 0.50–0.92), ADT + DOC (HR = 0.73, 95% CI: 0.62–0.86), and ADT + ENZ (HR = 0.64, 95% CI: 0.42–0.99) prolonged OS compared to ADT monotherapy, whereas ADT + RT (HR = 1.1, 95% CI: 0.92–1.2) did not demonstrate a survival advantage. Ranking results revealed that ADT + AAP was the best therapeutic option (62.28%) for the high-volume subgroup. No statistically significant differences were observed in the comparisons of the included combined systemic therapies, while ADT + RT was inferior to the other regimens in this subgroup.

In the low-volume disease subgroup, all combined treatments showed an OS benefit over ADT monotherapy, but statistical significance was only observed for ADT + ENZ (HR = 0.38, 95% CI: 0.21–0.69) and ADT + RT (HR = 0.68, 95% CI: 0.54–0.87). The ranking results revealed that ADT + ENZ was the best option (78.90%) for low-volume disease, although the benefit was non-significant. With the exception of ADT + ENZ vs. ADT + DOC (HR = 0.47, 95% CI: 0.25–0.89), none of the comparisons between the combined therapies yielded significant results.

### Subgroup Analysis of Gleason Score

**Figure 7:**
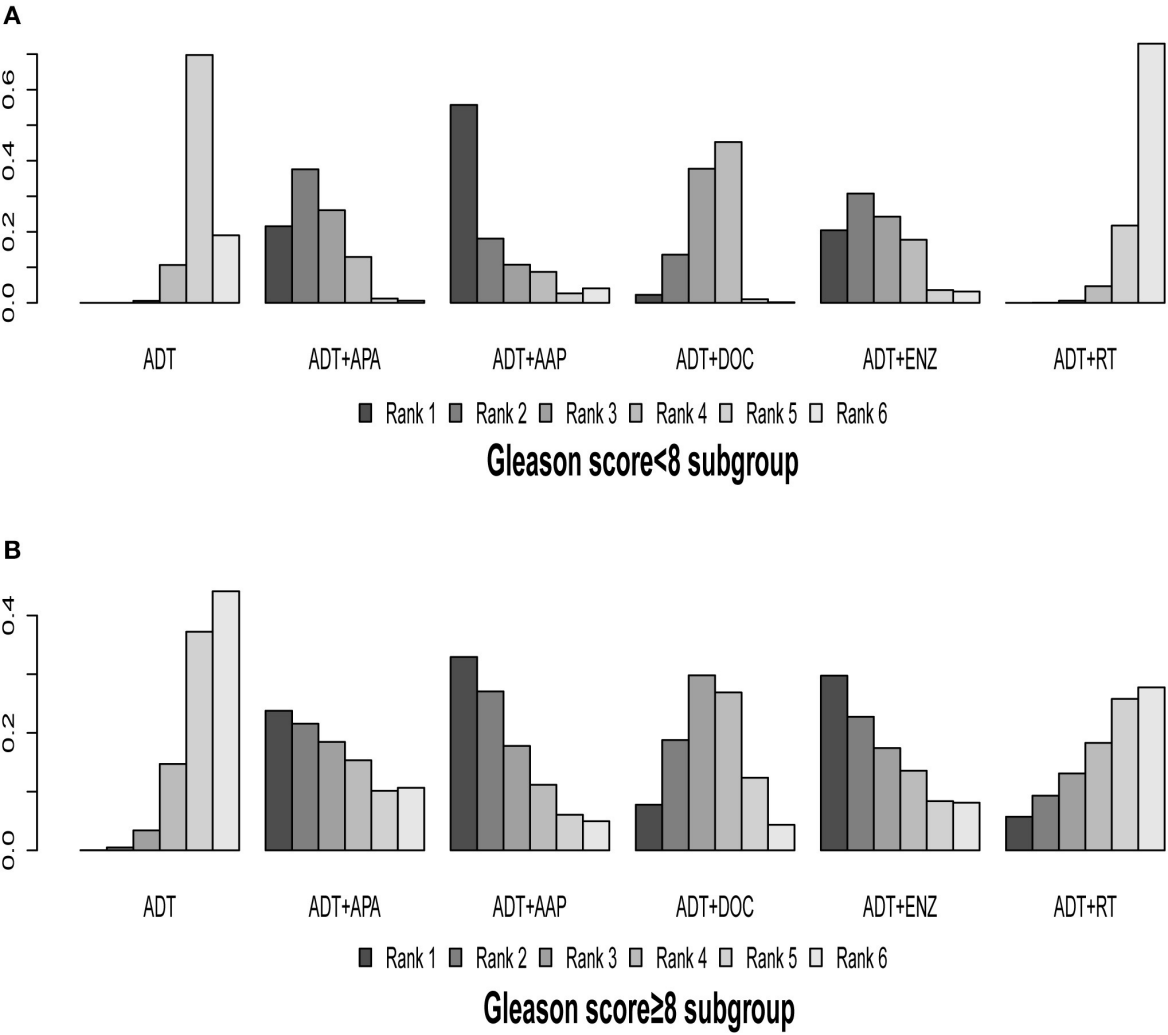
Ranking plot based on the probabilities of interventions in patients with different Gleason scores: **(A)** Gleason score <8 subgroup; **(B)** Gleason score ≥8 subgroup. The height of every column refers to the probabilities of the rank. The actual outcome being measured in this figure was overall survival.

The ENZAMET, TITAN, GETUG-AFU-15, CHAARTED, STAMPEDE-DOC arm, LATITUDE, and STAMPEDE-RT arm trials were included in this subgroup analysis; ARCHES and STAMPEDE-ABI arm trials were excluded because relevant outcome data were lacking. The results of indirect comparisons are shown in Table 2 and the ranking results are presented in Figure 7, Table 3.

In patients with a Gleason score <8, ADT + APA (HR = 0.56, 95% CI: 0.33–0.95) and ADT + DOC (HR = 0.71, 95% CI: 0.54–0.92) showed superiority over ADT monotherapy; similar trends were observed for ADT + AAP (HR = 0.44, 95% CI: 0.15–1.30) and ADT + ENZ (HR = 0.59, 95% CI: 0.30–1.2), although the differences were non-significant. ADT + RT (HR = 1.1, 95% CI: 0.84–1.50) was inferior to ADT monotherapy but without statistical significance. ADT + AAP (56.04%) was the highest-ranking combined treatment regimen.

In patients with a Gleason score ≥8, all of the combined treatments were superior to ADT monotherapy; however, the differences were not statistically significant. ADT + AAP (32.98%) ranked highest (HR = 0.65, 95% CI: 0.67–1.30) among the combined regimens.

Only two comparisons of combined therapies (ADT + RT vs. ADT + APA [HR = 2.0, 95% CI: 1.1–3.7] and ADT + RT vs. ADT + DOC [HR = 1.6, 95% CI: 1.1–2.4]) yielded statistically significant results, both in the Gleason <8 subgroup.

### Results of Convergence, Inconsistency, Publication Bias, and Heterogeneity Analyses

The potential scale reduction factor was limited to 1, reflecting good convergence in this analysis. The funnel plot of the included trials was near-symmetrical, indicating that there was no obvious publication bias. Given the lack of a closed loop in the network graph, the inconsistency assessment was not applicable to our study. The heterogeneity analysis for the whole network generated an *I*^2^-value of 35.54%, indicating that there was no significant heterogeneity. The full results of the convergence, inconsistency, publication bias, and heterogeneity analyses can be found in Supplementary Material 3.

## Discussion

The present study analyzed 10 high-quality, large-scale clinical trials involving 11,194 patients. All of the combined treatment regimens improved OS compared to ADT monotherapy, with ADT + ENZ ranking highest (although this result lacked statistical significance).

A strong association between PSA response and OS has been observed in *post-hoc* analyses of several large-scale clinical trials (18, 19). Our results showed that all combined treatment approaches except for ADT + RT were superior to ADT in the PSA-PFS analysis. However, the ranking results of PSA-PFS indicated that ADT + APA was the optimal regimen, which was inconsistent with the ranking results for OS. To address this discrepancy, we carefully examined the included trials and found that PSA-PFS survival data from the ENZAMET trial, unlike the stratified OS data, did not eliminate the influence of upfront docetaxel, which could explain the observed difference between the OS and PSA-PFS ranking results.

Both SSE and pain negatively affect the QoL and performance status of PC patients, thereby undermining the survival benefit of treatments (20, 21). Owing to a lack of data, only three combined regimens were included in our analyses of SSE and pain progression. Nonetheless, we found that all three regimens prolonged the time to SSE as well as the pain progression, whereas comparisons of different combined treatment regimens were equivocal due to a lack of statistical significance. Prolonging the time to initiate cytotoxic chemotherapy can reduce the frequency of severe adverse events and improve patients' QoL (22–24). In our analysis, both ADT + AAP and ADT + APA prolonged the time to chemotherapy compared to ADT monotherapy, with the ADT + APA regimen being the optimal therapy based on ranking results.

The CHAATERD and STAMPEDE trials demonstrated the superiority of ADT + DOC in the treatment of the high-volume disease subgroup of mHSPC (12, 17). Our subgroup analysis of high-volume mHSPC patients showed that all combined systemic therapies were superior to ADT monotherapy. Meanwhile, combining RT with ADT did not confer an additional survival advantage over ADT monotherapy in these patients. In accordance with the results of the CHAARTED trial, the benefits of ADT + APA, ADT + AAP, and ADT + DOC were more robust in the high-volume subgroup than in the low-volume subgroup. Recent studies have revealed the genomic basis of the greater responsiveness to treatment in the high-volume subgroup by sequencing, which showed that samples derived from these patients had more copy number alterations and a higher rate of *NOTCH* gene alterations than those from patients with low-volume disease (25). Alterations in the *NOTCH* gene have been linked to DOC resistance (26), which could explain our finding that other combined therapies were more effective than ADT + DOC in the high-volume subgroup.

A low-volume disease status is considered as an intermediate state between localized and disseminated metastatic PC. In our analysis, we did not distinguish between low-volume and oligometastatic HSPC as there is an overlap in their features that make them difficult to distinguish from each other. The status of the primary tumor site is an important prognostic factor in patients with oligometastatic disease. Findings from an animal model have indicated that the primary tumor secretes osteopontin into the circulation, promoting distal site metastasis (27). Multiple clinical trials and retrospective studies have also demonstrated that local therapy in HSPC patients slows disease progression and prolongs survival (8, 9, 28). Other advantages of treating primary PC include alleviating local symptoms as well as reducing the risk of adverse events caused by long-term systemic treatments (29). Thus, the management of the primary cancer site is critical for improving the outcome of mHSPC. Our results revealed that all combined therapies were superior to ADT monotherapy in terms of survival benefit. Local RT complementary to ADT showed some advantage over several combined systemic regimens; however, the lack of statistical significance limits the reliability of these findings. Data from ongoing clinical trials (SWOG S1802 [NCT03678025], TROMBONE [ISRCTN 15704862], g-RAMPP [NCT02454543], and SIMCAP [NCT03456843]) could provide more insight into the benefits of local therapies combined with systemic treatment. Although ADT + ENZ was identified as the most effective regimen in the low-volume subgroup, comparisons between ADT + ENZ and the other combined treatments did not yield significant results. Therefore, our findings should be interpreted with caution.

Gleason score is the most important predictor of PC prognosis although its utility for treatment decision-making is debated. The results of our study showed that all combined systemic treatment approaches were superior to ADT monotherapy in each Gleason score subgroup and that ADT + AAP conferred the greatest survival advantage irrespective of Gleason score. We also found that concurrent RT did not provide an additional benefit compared to ADT monotherapy in the Gleason score subgroup analysis, although the reliability of our results was limited by several factors. Firstly, there was considerable biological heterogeneity in both the Gleason ≥8 and Gleason <8 subgroups, which could undermine our conclusions (30). In order to reduce heterogeneity, the Pathologic Grading System of the International Society of Urological Pathology has defined Gleason score 8 (Grade Group [GG] 4) and Gleason score 9–10 (GG 5) disease as distinct entities while also distinguishing between the Gleason 3+4 and Gleason 4+3 subgroups (31); however, this grading system has not been adopted in all current clinical trials. Furthermore, survival data from the ENZAMET study were imprecise in this subgroup analysis as they did not exclude patients who received upfront docetaxel, which could have influenced our analyses. Despite these limitations, ours is the only comparative analysis of treatment efficacy in mHSPC patients with different Gleason scores.

To our knowledge, this is the first study comparing the efficacy of ADT combined with systemic treatments or RT in mHSPC patients. The results provide a basis for developing targeted and individualized management strategies for mHSPC patients; they also clarify the role and outcomes of local RT in the treatment of mHSPC, and identify the subgroup of patients that is most likely to benefit from this treatment modality.

Our study had several limitations. Firstly, eligible clinical trials evaluating radical prostatectomy complementary to ADT were not available, which made it impossible to compare the efficacy of ADT plus prostatectomy with other combined treatment options. Secondly, the trials related to RT had several inherent shortcomings. For example, the RT doses delivered to the primary tumor in both the HORRAD and STAMPEDE-H arm (ADT + RT) trials were lower than the current standard of care; in the latter study, the RT was 36 Gy in 6 fractions or 55 Gy in 20 fractions, which are lower than the 57-Gy regimen that was shown to be inferior in the CHHiP trial (32). Another limitation related to the HORRAD trial is that it was considered as underpowered from the standpoint of metastatic burden (33). Moreover, the RT used in studies included in our analysis was limited to the prostate, with no trials evaluating the efficacy of metastasis-directed therapy using stereotactic body radiation therapy (SBRT). Although our literature search identified clinical trials of SBRT in oligometastatic PC patients (34), they did not meet our inclusion criteria and were therefore excluded. Thirdly, the lack of subgroup and patient-level data in the included trials prevented us from arriving at more reliable conclusions. Finally, the lack of direct comparisons between different combined treatment approaches in mHSPC patients limits the accuracy of our results. Data from ongoing head-to-head clinical trials (e.g., PEACE-1 [NCT01957436]) are needed to validate our findings.

In summary, ADT + RT demonstrated superiority over ADT monotherapy in our analysis of OS and the low-volume subgroup of mHSPC patients. Furthermore, ADT + RT showed comparable efficacy to most combined systemic treatment regimens in the low-volume subgroup. The combined systemic therapies showed a significant advantage over ADT monotherapy in all comparisons performed in this study, with ADT + ENZ identified as the optimal treatment in most cases. Based on limited data, we also showed that patients receiving combined therapies experienced less of a decline in QoL compared to those treated with ADT monotherapy. Based on these findings, the selection of an appropriate treatment approach for mHSPC patients by the physician should be made based on discussions regarding potential toxicities as well as the duration and cost of treatment.

## Data Availability Statement

All datasets generated for this study are included in the article/Supplementary Material.

## Author Contributions

YW and ZW: study concept and design. YW and HG: acquisition of data and analysis and interpretation of data. YW: drafting of the manuscript. JT, HW, ZH, and ZW: critical revision of the manuscript. YW and JW: statistical analysis. ZW: obtaining funding. All authors contributed to the article and approved the submitted version.

## Conflict of Interest

The authors declare that the research was conducted in the absence of any commercial or financial relationships that could be construed as a potential conflict of interest.
