# Supplementary material for: Comparative Efficacy of Combined Radiotherapy, Systemic Therapy, and Androgen Deprivation Therapy for Metastatic Hormone-Sensitive Prostate Cancer: A Network Meta-Analysis and Systematic Review
Source: Front Oncol. 2020 Oct 20;10:567616. doi: 10.3389/fonc.2020.567616 (PMC7606969; doi:10.3389/fonc.2020.567616)
Supplement: Supplementary file 1 [file Data_Sheet_1.ZIP › Supplementary_Material 2_PRISMA flow diagram.pdf]

PRISMA 2009 Flow Diagram

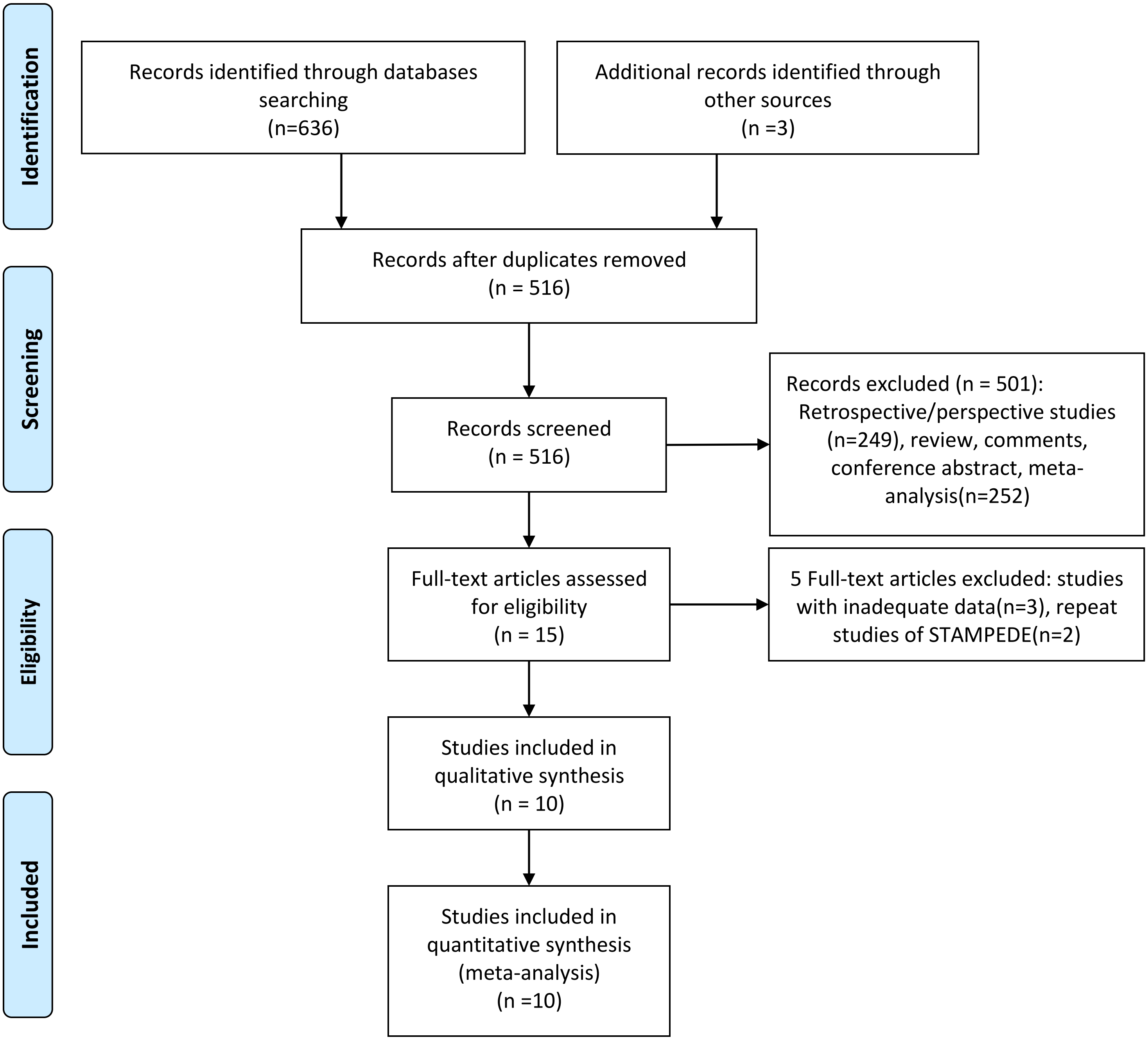

From: Moher D, Liberati A, Tetzlaff J, Altman DG, The PRISMA Group (2009). *P*referred *R*eporting *I*tems for *S*ystematic Reviews and *M*eta-*A*nalyses: The PRISMA Statement. PLoS Med 6(7): e1000097. doi:10.1371/journal.pmed1000097

For more information, visit www.prisma-statement.org.
