# Supplementary material for: Comparative Efficacy of Combined Radiotherapy, Systemic Therapy, and Androgen Deprivation Therapy for Metastatic Hormone-Sensitive Prostate Cancer: A Network Meta-Analysis and Systematic Review
Source: Front Oncol. 2020 Oct 20;10:567616. doi: 10.3389/fonc.2020.567616 (PMC7606969; doi:10.3389/fonc.2020.567616)

Supplementary Material

**1. Results of convergence analysis**

Note: “1” refers to " androgen deprivation therapy "; “2” refers to "androgen deprivation therapy + apalutamide"; “3” refers to " androgen deprivation therapy + abiraterone and prednisolone"; “4” refers to " androgen deprivation therapy + docetaxel"; “5” refers to " androgen deprivation therapy + enzalutamide"; “6” refers to " androgen deprivation therapy + radiotherapy ".

**Supplementary Figure 1. Brooks-Gelman-Rubin plot**


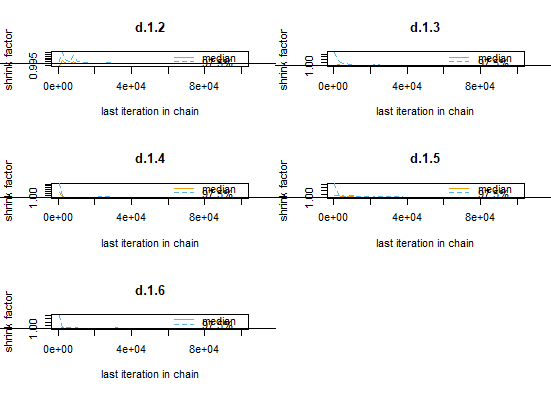


**Supplementary Figure 2. Trace plot and density plot**


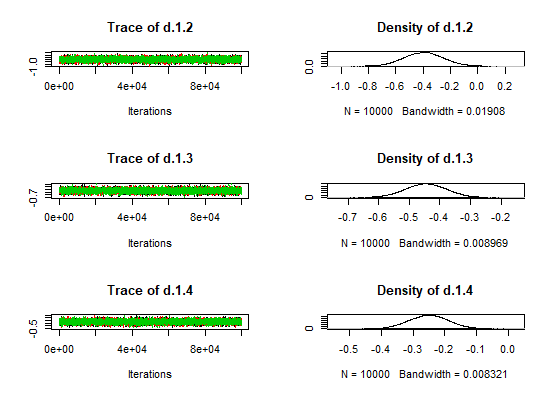

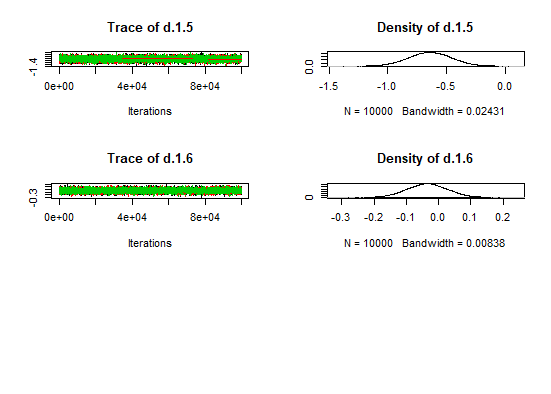


**2. Results of heterogeneity analysis**

Note: “1” refers to " androgen deprivation therapy "; “2” refers to "androgen deprivation therapy + apalutamide"; “3” refers to " androgen deprivation therapy + abiraterone and prednisolone"; “4” refers to " androgen deprivation therapy + docetaxel"; “5” refers to " androgen deprivation therapy + enzalutamide"; “6” refers to " androgen deprivation therapy + radiotherapy ".

**2.1 Overall Survival**

(1) Analysis of heterogeneity

=========================

Per-comparison I-squared:

-------------------------

|  | t1 | t2 | i2.pair | i2.cons | incons.p |
| --- | --- | --- | --- | --- | --- |
| 1 | 2 | NA | NA | NA |  |
|  | 1 | 3 | 0.00000 | 0.00000 | NA |
|  | 1 | 4 | 52.18910 | 52.12437 | NA |
|  | 1 | 5 | NA | NA | NA |
|  | 1 | 6 | 44.63317 | 44.92191 | NA |

Global I-squared:

-------------------------

i2.pair i2.cons

1 37.34687 37.40508

(2) Results of tau value calculation (using random effect model, s.d.d in this analysis refers to tau value)

Results on the Log Hazard Ratio scale

Iterations = 5001:25000

Thinning interval = 1

Number of chains = 3

Sample size per chain = 20000

1. Empirical mean and standard deviation for each variable,

plus standard error of the mean:

|  | Mean | SD | Naïve SE | Time-series SE |
| --- | --- | --- | --- | --- |
| d.1.2 | -0.3960 | 0.2276 | 0.0009290 | 0.0021598 |
| d.1.3 | -0.4510 | 0.1442 | 0.0005887 | 0.0009137 |
| d.1.4 | -0.2635 | 0.1218 | 0.0004971 | 0.0009746 |
| d.1.5 | -0.6422 | 0.2523 | 0.0010301 | 0.0028239 |
| d.1.6 | -0.0111 | 0.1441 | 0.0005882 | 0.0009884 |
| **sd.d** | **0.1377** | **0.1136** | **0.0004638** | **0.0026634** |

2. Quantiles for each variable:

|  | 2.5% | 25% | 50% | 75% | 97.5% |
| --- | --- | --- | --- | --- | --- |
| d.1.2 | -0.85449 | -0.52479 | -0.39705 | -0.26687 | 0.06042 |
| d.1.3 | -0.75503 | -0.52218 | -0.45087 | -0.37906 | -0.15620 |
| d.1.4 | -0.51937 | -0.32580 | -0.26175 | -0.19869 | -0.01926 |
| d.1.5 | -1.14443 | -0.79380 | -0.64330 | -0.49025 | -0.14605 |
| d.1.6 | -0.29371 | -0.08479 | -0.01806 | 0.05773 | 0.30109 |
| sd.d | 0.00554 | 0.05449 | 0.10900 | 0.18877 | 0.44619 |

-- Model fit (residual deviance):

| Dbar | pD | DIC |
| --- | --- | --- |
| 9.373893 | 7.426614 | 16.800508 |

9 data points, ratio 1.042, I^2 = 15%

**3. Results of publication bias**

**Supplementary Figure 3. The Funnel Plot of Enrolled Trials**

Note: “1” refers to " androgen deprivation therapy "; “2” refers to "androgen deprivation therapy + apalutamide"; “3” refers to " androgen deprivation therapy + abiraterone and prednisolone"; “4” refers to " androgen deprivation therapy + docetaxel"; “5” refers to " androgen deprivation therapy + enzalutamide"; “6” refers to " androgen deprivation therapy + radiotherapy ".


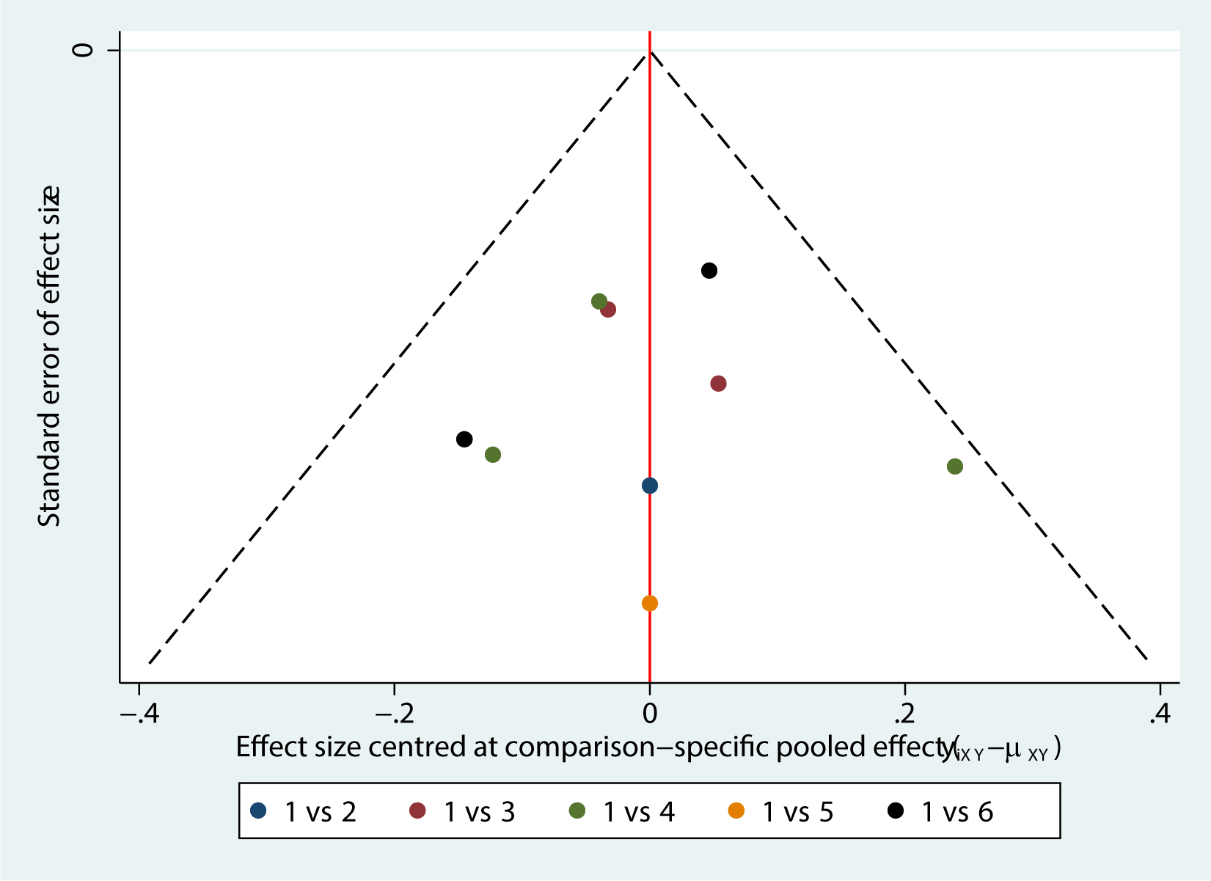

Supplement: Supplementary file 1 [file Data_Sheet_1.ZIP › Supplementary_Material 3_Results of convergence, heterogeneity analysis and publication bias.docx]
