# Supplementary material for: Comparative Efficacy of Combined Radiotherapy, Systemic Therapy, and Androgen Deprivation Therapy for Metastatic Hormone-Sensitive Prostate Cancer: A Network Meta-Analysis and Systematic Review
Source: Front Oncol. 2020 Oct 20;10:567616. doi: 10.3389/fonc.2020.567616 (PMC7606969; doi:10.3389/fonc.2020.567616)
Supplement: Supplementary file 1 [file Data_Sheet_1.ZIP › Supplementary_Material 1_Results of full search strategy of PubMed database.docx]

Supplementary Material

**Supplementary data 1. Full search strategy of PubMed/MEDLINE database**

1 ((((((((((((((((Prostate Neoplasms[Title/Abstract]) OR Neoplasms, Prostate[Title/Abstract]) OR Neoplasm, Prostate[Title/Abstract]) OR Prostate Neoplasm[Title/Abstract]) OR Neoplasms, Prostatic[Title/Abstract]) OR Neoplasm, Prostatic[Title/Abstract]) OR Prostatic Neoplasm[Title/Abstract]) OR Prostate Cancer[Title/Abstract]) OR Cancer, Prostate[Title/Abstract]) OR Cancers, Prostate[Title/Abstract]) OR Prostate Cancers[Title/Abstract]) OR Cancer of the Prostate[Title/Abstract]) OR Prostatic Cancer[Title/Abstract]) OR Cancer, Prostatic[Title/Abstract]) OR Cancers, Prostatic[Title/Abstract]) OR Prostatic Cancers[Title/Abstract]) OR Cancer of Prostate[Title/Abstract]

2 "Prostatic Neoplasms"[Mesh]

3 (#1) OR #2

4 "Abiraterone Acetate"[Mesh]

5 (((((Abiraterone[Title/Abstract]) OR 17-(3-pyridyl)-5,16-androstadien-3beta-acetate[Title/Abstract]) OR Zytiga[Title/Abstract]) OR CB 7630[Title/Abstract]) OR CB-7630[Title/Abstract]) OR CB7630[Title/Abstract]

6 (#4) OR #5

7 "Docetaxel"[Mesh]

8 (((((((((((Docetaxel Trihydrate[Title/Abstract]) OR Docetaxol[Title/Abstract]) OR Docetaxel Hydrate[Title/Abstract]) OR Taxoltere Metro[Title/Abstract]) OR RP 56976[Title/Abstract]) OR RP-56976[Title/Abstract]) OR RP56976[Title/Abstract]) OR Taxotere[Title/Abstract]) OR Docetaxel Anhydrous[Title/Abstract]) OR N-Debenzoyl-N-tert-butoxycarbonyl-10-deacetyltaxol[Title/Abstract]) OR N Debenzoyl N tert butoxycarbonyl 10 deacetyltaxol[Title/Abstract]) OR NSC 628503[Title/Abstract]

9 (#7) OR #8

10 enzalutamide[Title/Abstract]

11 apalutamide[Title/Abstract]

12 "Radiotherapy"[Mesh]

13 ((((Radiotherapies[Title/Abstract]) OR (Radiation Therap*[Title/Abstract])) OR (Radiation Treatment*[Title/Abstract])) OR (targeted Radiotherap*[Title/Abstract])) OR (Targeted Radiation Therap*[Title/Abstract])

14 (#12) OR #13

15 (#3) AND #6

16 (#3) AND #9

17 (#3) AND #10

18 (#3) AND #11

19 (#3) AND #14

20 ((((#13) OR #15) OR #16) OR #17) OR #18

21 (randomized controlled trial [pt] OR controlled clinical trial [pt] OR randomized [tiab] OR placebo [tiab] OR clinical trials as topic [mesh: noexp] OR randomly [tiab] OR trial [ti]) NOT (animals [mh] NOT humans [mh]))

22 (#20) AND #21

23 metasta*[Title/Abstract]

24 (#22) AND #23

25 (hormone[Title/Abstract]) OR (castrat*[Title/Abstract])

26 (sensitive[Title/Abstract]) OR (naive[Title/Abstract])

27 #25 AND #26

28 #24 AND #27
